# Serum vitamin C levels and their correlation with chronic kidney disease in adults: a nationwide study

**DOI:** 10.1080/0886022X.2023.2298079

**Published:** 2024-01-08

**Authors:** Chunli Wang, Jili Zhao, Qiaoqiao Zhou, Jing Li

**Affiliations:** Department of General Internal Medicine, Sichuan Cancer Hospital and Institute, Sichuan Cancer Center, School of Medicine, University of Electronic Science and Technology of China, Chengdu, Sichuan, China

**Keywords:** Vitamin C, oxidative stress, albuminuria, eGFR, chronic kidney disease

## Abstract

**Introduction:**

Inflammation and oxidative stress play significant roles in the development of chronic kidney disease (CKD). Given the recognized antioxidant properties of vitamin C, our study aimed to explore the correlation between CKD and serum vitamin C levels.

**Methods:**

Data were gathered from the 2017–2018 National Health and Nutrition Examination Survey. Participants below 18 years of age, pregnant individuals, those lacking essential data for CKD diagnosis, or individuals with incomplete serum vitamin C data were excluded. Subgroup and weighted multivariable logistic regression analyses were performed to assess the potential correlation between serum vitamin C and CKD.

**Results:**

Our study comprised 4969 participants, revealing an overall CKD prevalence of 15.0%. The results indicated that individuals with reduced serum vitamin C levels were more likely to be male, possess lower educational attainment, have a diminished poverty-income ratio, engage in heavy drinking, and be current smokers. Additionally, they exhibited a higher prevalence of obesity and diabetes. Significantly, participants in the third quartile group experienced a 37.0%, 47.0%, and 46.6% decrease in the risk of developing albuminuria, low estimated glomerular filtration rate (eGFR), and CKD, respectively. Subgroup analysis demonstrated that individuals between 65 and 80 years of age showed a statistically reduced risk of developing CKD and low eGFR when their serum vitamin C levels fell in the third and fourth quartile groups.

**Conclusions:**

Our findings reveal a correlation between elevated serum vitamin C levels and a decreased risk of developing albuminuria, low eGFR, and CKD. Appropriately increasing serum vitamin C levels may hold promise in protecting renal function, particularly among older individuals.

## Introduction

Chronic kidney disease (CKD) represents a significant global public health challenge, affecting over 850 million individuals [1,2]. On a global epidemiological scale, the burden of CKD has notably escalated in the United States (US) [3]. Research indicates that the prevalence of CKD in the US exhibited a substantial growth rate between 2002 and 2016, surpassing that of other disorders. By 2017, the proportion of CKD patients in the US had reached 14.5%, accompanied by expenditures exceeding 120 billion dollars [3]. Timely identification and intervention concerning risk factors play a pivotal role in averting CKD and improving renal outcomes. Risk factors for CKD in the general population encompass proteinuria, metabolic syndrome, diabetes, hypertension, advanced age, nephrotoxic agents, among others. Notably, proteinuria is recognized as both an initiating and perpetuating factor for CKD [4]. Meta-analyses have unequivocally established a link between proteinuria and heightened all-cause and cardiovascular mortality risk [5]. Presently, conventional CKD treatment employs a multifaceted approach involving blood pressure and blood glucose management, proteinuria control using renin-angiotensin system (RAS) blockers, as well as the mitigation of obesity and avoidance of nephrotoxic substances [6]. Nevertheless, therapeutic options for CKD remain somewhat constrained.

Oxidative stress plays a significant role in the risk factors associated with CKD. Even in well-controlled hypertensive patients treated with RAS blockers, research indicates that proteinuria is associated with systemic damage due to oxidative stress [7]. The advent of sodium-glucose cotransporter 2 inhibitors, a novel class of antihyperglycemic drugs, has shown promise in slowing or reversing the progression of proteinuria and reducing the risk of reactive oxygen species (ROS) damage to renal tubules [8]. It is widely acknowledged that oxidative stress and inflammation substantially contribute to CKD development [9–11]. Oxidative stress occurs when ROS surpasses the body’s antioxidant defenses, leading to cellular damage, including damage to DNA, proteins, and lipids. The mitochondrial respiratory chain serves as the primary intracellular source of ROS [12]. Due to the kidney’s high mitochondrial density and oxygen demands, oxidative damage is prone to occur, thereby expediting the progression of kidney disease [13]. Notably, CKD and dialysis patients bear an increased burden of oxidative stress due to diminished antioxidant defenses and heightened pro-oxidant activity [11].

Vitamin C, a ubiquitous non-enzymatic antioxidant naturally present in a diverse range of fruits and vegetables [14], emerges as a potential candidate for intervention. Given the elevated levels of oxidative stress in CKD patients, antioxidant therapy involving vitamin C presents itself as a viable strategy for enhancing outcomes. However, research on the correlation between vitamin C and CKD remains limited. Therefore, the objective of this study was to explore the correlation of vitamin C levels in serum and CKD. We aim to investigate whether elevating serum levels through additional vitamin C supplementation, whether from dietary sources or dietary supplements, can improve the prognosis for kidney disease patients and ultimately confer clinical benefits upon them.

## Materials and methods

### Study population

In this study, the data utilized were derived from the National Health and Nutrition Examination Survey (NHANES). NHANES, a cross-sectional survey, is designed with the overarching objective of achieving national representativeness among non-institutionalized American civilians. This comprehensive survey encompasses a spectrum of data, including demographic particulars, physical examinations, laboratory tests, health questionnaires, and nutritional information.

**Figure 1.**
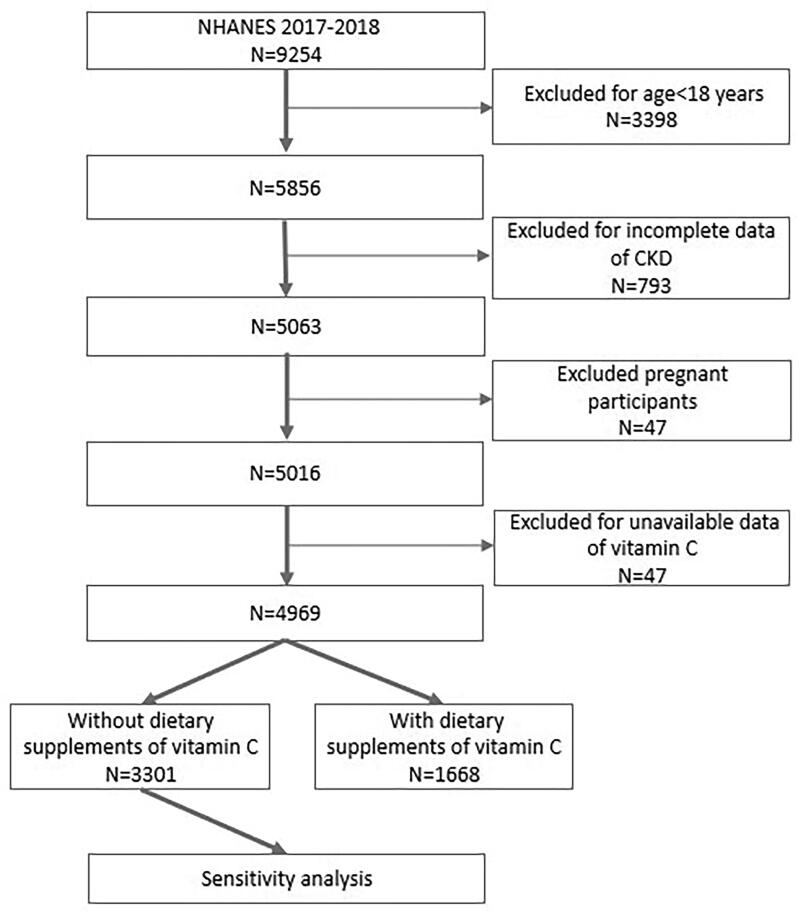
A flowchart showing the sample selection from NHANES 2017–2018.

This study analyzed data collected during the NHANES 2017–2018 cycle, a period in which records pertaining to both serum vitamin C concentration and the urinary albumin-to-creatinine ratio (ACR) were collected. Initially, a cohort comprising a total of 9254 individuals participated in the 2017–2018 cycle. However, an exclusionary process was applied, leading to the removal of individuals below 18 years of age (*n* = 3398) from the analytical framework. Simultaneously, those lacking sufficient data for CKD diagnosis (*n* = 793) and pregnant individuals (*n* = 47) were systematically excluded. Furthermore, participants devoid of data concerning serum vitamin C were also systematically omitted from consideration (*n* = 47). Consequently, our analytical focus converged on a definitive cohort comprising 4969 participants, as delineated in Figure 1. It is imperative to underscore that all participating individuals provided explicit written informed consent, and the ethical review board at the National Center for Health Statistics granted approval for the NHANES protocols.

### Primary exposure

The exposure variable in this study was serum vitamin C concentration. The quantification of vitamin C in the serum was accomplished utilizing isocratic ultra-high performance liquid chromatography, incorporating electrochemical detection set at 450 mV (with a range of 200 nA). Preceding analysis, specimens underwent acidification, stabilization, and subsequent freezing at −70 °C. The laboratory methodology for ascertaining serum vitamin C levels has been extensively expounded upon elsewhere [15]. In total, 6740 participants aged ≥6 years met the stipulated criteria in this examination and underwent the measurement of vitamin C levels in the serum, following the aforementioned protocols. The resultant serum vitamin C concentrations were stratified into quartiles, delineated as follows: ≥67.6, 50.3–67.6, 30.4–50.3, and <30.4 µmol/L.

The variable under investigation in this study was the presence of chronic kidney disease (CKD), dichotomized as either present or absent. In accordance with the Kidney Disease: Improving Global Outcomes 2012 recommendations, the identification of CKD was predicated on the presence of either a diminished eGFR (< 60 mL/min/1.73 m^2^) or the manifestation of albuminuria [16]. The eGFR was derived utilizing the Chronic Kidney Disease Epidemiology Collaboration (CKD-EPI) equation, which factors in variables such as gender, age, and serum creatinine. In contrast to the Modification of Diet in Renal Disease (MDRD) equation, the CKD-EPI equation demonstrates superior accuracy and diminished deviations [17]. Albuminuria was specifically delineated as a urinary ACR ≥ 30 mg/g [18].

### Covariates

The present study incorporated various covariates to comprehensively address factors that may potentially influence the correlation between CKD and serum vitamin C. Primarily, demographic factors, including race, gender, age, family income, and education level, were taken into consideration. Age was categorized into three groups: 65–80, 45–64, and 18–44 years. Race was classified as non-Hispanic Asian, non-Hispanic Black, non-Hispanic White, Hispanic, and other races. Education level was stratified as ‘more than high school,’ ‘high school,’ and ‘less than high school.’ Family income, represented by the ‘family income to poverty ratio,’ was grouped as >1.8, 1.3–1.8, and <1.3 [19]. Additionally, body mass index (BMI) was stratified into categories of obesity (≥30.0 kg/m^2^), overweight (25.0–30.0 kg/m^2^), and under/normal weight (<25.0 kg/m^2^) [20]. Smoking status was determined through self-reported data and categorized as current smoker, former smoker, and nonsmoker. Participants were divided into groups according to the 2018 US Physical Activity Guidelines: inactive, less active, and active [21]. Alcohol consumption was categorized in accordance with the National Institute guidelines on Alcohol Abuse and Alcoholism as heavy (women: ≥2 drinks/day or men: ≥3 drinks/day), moderate (women: 1 drink/day or men: 1–2 drinks/day), and none [22]. Dietary vitamin C intake from food was segmented into quartiles: < 25.6 mg/d, 25.6–56.7 mg/d, 56.7–106.7 mg/d, and ≥106.7 mg/d. Dietary supplements of vitamin C, originating from various sources taken in the past 30 days, were classified into daily doses: 0, 1–60 mg, 61–120 mg, 121–500 mg, or ≥500 mg [15]. According to the Clinical Practice Guideline (2017) from the American College of Cardiology and American Heart Association, hypertension was confirmed if the participant had an average diastolic blood pressure ≥80 mmHg and/or an average systolic blood pressure ≥130 mmHg, or a hypertension history or reported antihypertensive medicine utilization [23]. Diabetes was characterized by a history of diabetes, hypoglycemic medicine utilization, or a hemoglobin A1c (HbA1c) level ≥6.5% [24]. Low-density lipoprotein cholesterolemia was defined as a serum low-density lipoprotein cholesterol level ≥ 4.14 mmol/L [25]. Additional baseline variables encompassed aspirin use (yes/no), high-density lipoprotein cholesterol levels (mmol/L), high-sensitivity C-reactive protein (hs-CRP) levels (mg/L), total cholesterol levels (mmol/L), serum creatinine levels (µmol/L), urine protein levels (mg/L), serum uric acid levels (µmol/L), blood urea nitrogen levels (mmol/L), hemoglobin levels (g/dl), and HbA1c levels (%). Further details can be found at www.cdc.gov/nchs/nhanes/.

## Statistical analysis

In accordance with guidelines established by the Centers for Disease Control and Prevention, all statistical analyses were performed. Continuous variables were presented as weighted mean ± standard deviation and analyzed using weighted linear regression. Categorical variables were delineated as weighted percentages, accompanied by corresponding 95% confidence intervals (CI), and analyzed using the chi-square test. Multivariable logistic regression models were employed to examine the correlation of CKD and serum vitamin C levels. Confounders were chosen among variables causing the exposure variable or outcome variables, or both [26]. Covariates were selected as adjustment factors based on their significant impact on outcome variables or a change in the regression coefficient exceeding 10%. Supplementary Table S1 displays the associations between each covariate and outcome variables. Model 1 did not involve any covariate adjustments. Model 2 incorporated adjustments solely for race, age, and gender. The final model embraced a comprehensive set of variables: race, age, gender, poverty income ratio (PIR), education level, hypertension, diabetes, BMI, physical activity, aspirin use, hs-CRP, smoking status, alcohol consumption, dietary vitamin C derived from food, and dietary supplements of vitamin C.

Subgroup analysis was conducted to assess the impact of race, gender, and age on the study’s outcomes. To account for the potential influence of participants taking additional vitamin C supplements, leading to higher serum vitamin C concentrations, a sensitivity analysis was performed. In this analysis, participants with dietary vitamin C supplements were excluded to assess the stability of the results.

R software (http://www.R-project.org, The R Foundation, Austria), STATA 16.0 (StataCorp, College Station, TX, USA), and Empowerstats (http://www.empowerstats.com, X&Y Solutions, Inc, CA, USA) were employed for all statistical analyses. The significance threshold was set at *p*<.05 for all analyses.

## Results

### Baseline characteristics of participants

Totally, this study included 4969 participants, with a gender distribution of 51.2% female and 48.8% male, exhibiting an average age of 47.5 ± 17.7 years. The comprehensive characteristics of the participants, stratified by quartiles of serum vitamin C concentration, are delineated in Table 1. In the participant cohort, 15.0% exhibited CKD. Statistically significant disparities across all outcome measures (all *p* < .05) were discernible among the four quartiles of serum vitamin C levels. Participants with elevated serum vitamin C concentrations demonstrated an inclination toward higher educational attainment, non-Hispanic White ethnicity, female gender, and elevated PIR. Conversely, those with diminished serum vitamin C levels tended to possess lower educational qualifications, male gender, lower PIR, and an increased likelihood of being current smokers and heavy drinkers. This subgroup also manifested a higher obesity and diabetes prevalence. Furthermore, our study revealed an inverse correlation between serum vitamin C concentration and indicators such as urine protein, ACR, hsCRP levels, and HbA1c. However, no statistically significant trends were observed concerning age and levels of physical activity.

**Table 1.** Baseline characteristics of participants (*n* = 4969) according to vitamin C (quartiles 1–4, umol/L).

	Total (*n* = 4969)	Quartiles 1 (<30.4) (*n* = 1242)	Quartiles 2(30.4–50.3) (*n* = 1236)	Quartiles 3 (50.3–67.6) (*n* = 1239)	Quartiles 4 (≥67.6) (*n* = 1252)	*p*
Age (years)	47.5 ± 17.7	46.2 ± 16.8	45.8 ± 17.1	46.6 ± 17.2	51.3 ± 18.8	<.001
18–44	45.0 (43.0–47.1)	46.6 (42.5–50.7)	49.9 (45.7–54.1)	47.8 (43.5–52.1)	36.6 (32.7–40.6)	
45–64	26.7 (24.8–28.8)	28.5 (24.6–32.7)	26.0(22.1–30.2)	26.6 (22.7–30.8)	25.9 (22.1–30.1)	
65–80	28.2 (26.4–30.1)	25.0 (21.7–28.5)	24.1 (20.7–27.8)	25.7 (22.2–29.5)	37.5 (33.7–41.5)	
Urine protein (mg/L)	38.1 ± 278.6	61.0 ± 392.8	46.2 ± 353.7	25.4 ± 182.6	21.4 ± 69.8	<.001
UACR (mg/g)	35.6 ± 269.5	55.2 ± 374.6	40.9 ± 291.2	23.8 ± 246.4	23.7 ± 88.0	.007
SCr (umol/L)	77.7 ± 28.3	81.4 ± 41.0	79.9 ± 27.6	76.3 ± 19.8	73.7 ± 19.0	<.001
eGFR (ml/min/1.73 m^2^)	94.2 ± 22.3	93.9 ± 22.6	94.6 ± 23.0	96.5 ± 21.2	91.8 ± 22.2	<.001
HbA1C (%)	5.7 ± 0.9	5.8 ± 1.1	5.7 ± 0.9	5.6 ± 0.8	5.6 ± 0.8	<0.001
hs-CRP (mg/L)	3.8 ± 7.7	5.2 ± 9.7	4.2 ± 9.5	3.2 ± 5.5	2.8 ± 4.7	<.001
BUN (mmol/L)	5.3 ± 1.9	5.2 ± 2.1	5.3 ± 1.8	5.3 ± 1.7	5.4 ± 1.9	.004
SUA (umol/L)	320.2 ± 85.2	328.5 ± 88.5	332.0 ± 89.6	323.8 ± 82.7	298.4 ± 76.1	<.001
Hb (g/dL)	14.2 ± 1.5	14.4 ± 1.6	14.3 ± 1.5	14.2 ± 1.5	14.0 ± 1.3	<.001
TC (mmol/L)	4.9 ± 1.0	4.9 ± 1.1	4.9 ± 1.1	4.8 ± 1.0	4.9 ± 1.0	.008
HDL (mmol/L)	1.4 ± 0.4	1.3 ± 0.4	1.3 ± 0.4	1.4 ± 0.4	1.5 ± 0.4	<.001
Diabetes (%)						<.001
Yes	15.0 (13.6–16.5)	18.4 (15.5–21.8)	15.7 (13.1–18.8)	13.6 (11.1–16.6)	12.3 (10.0–15.1)	
Hypertension (%)						<.001
Yes	51.3 (49.2–53.4)	56.8 (52.6–60.9)	54.7 (50.5–58.9)	46.9 (42.6–51.2)	47.4 (43.3–51.5)	
LDL-C(%)						<.001
Yes	3.8 (3.1–4.7)	3.9 (2.6–5.9)	4.6 (3.1–6.6)	2.9 (1.9–4.6)	3.8(2.4–6.0)	
Dietary VC intake by food,(mg/d)						<.001
<25.6	24.3 (22.5–26.2)	45.6 (41.5–49.8)	22.5 (19.1–26.3)	16.6 (13.8–19.8)	12.9 (10.3–16.1)	
25.6–56.7	24.1 (22.3–26.0)	27.7 (24.2–31.5)	27.7 (24.0–31.8)	21.7 (18.3–25.5)	19.8 (16.7–23.4)	
56.7–106.7	22.8 (21.1–24.7)	13.9 (11.3–17.1)	26.6 (22.9–30.6)	23.1 (19.6–27.0)	27.9 (24.3–31.8)	
≥106.7	22.4 (20.6–24.2)	7.3 (5.5–9.5)	16.5 (13.9–19.5)	32.2 (28.1–36.5)	32.4 (28.6–36.3)	
Not recorded	6.4 (5.6–7.4)	5.5 (3.9–7.7)	6.7 (5.3–8.4)	6.5 (4.9–8.6)	7.0 (5.4–9.1)	
Daily dose of VC supplements, mg						<.001
None	63.5 (61.4–65.5)	87.3 (83.8–90.0)	69.2 (65.1–73.1)	59.1 (54.7–63.4)	39.9 (35.9–44.0)	
1–60	11.5 (10.1–12.9)	7.1 (5.0–10.2)	12.5 (10.1–15.5)	14.4 (11.4–18.0)	11.7 (9.4–14.5)	
61–120	11.6 (10.3–13.1)	3.4 (2.1–5.5)	10.6 (8.2–13.6)	14.2 (11.2–17.7)	17.9 (14.9–21.3)	
121–500	7.7 (6.6–9.0)	1.2 (0.7–2.2)	5.8 (3.7–8.8)	7.3 (5.4–9.9)	16.1 (13.1–19.6)	
≥500	5.8 (4.8–6.9)	1.0 (0.5–2.1)	1.9 (1.1–3.4)	5.0 (3.5–7.3)	14.5 (11.7–17.8)	
Gender (%)						<.001
Male	48.9 (46.7–51.0)	56.6 (52.5–60.6)	54.3 (50.1–58.4)	53.3 (49.0–57.6)	32.3 (28.5–36.3)	
Female	51.2 (49.0–53.3)	43.4 (39.4–47.6)	45.7 (41.6–49.9)	46.7 (42.5–51.0)	67.7 (63.7–71.5)	
Race (%)						<.001
Hispanic	16.3 (15.2–17.5)	11.9 (10.1–14.0)	22.1 (19.5–25.0)	17.5 (15.3–20.0)	14.4 (12.4–16.6)	
Non-Hispanic White	62.7 (60.9–64.5)	67.9 (64.6–71.1)	51.6 (47.4–55.7)	60.7 (56.9–64.4)	69.3 (66.2–72.3)	
Non-Hispanic Black	10.6 (9.8–11.4)	10.9 (9.5–12.5)	13.4 (11.6–15.3)	10.3 (8.9–12.0)	8.0 (6.8–9.5)	
Non-Hispanic Asian	5.6 (5.2–6.1)	3.4 (2.7–4.2)	7.2 (6.1–8.6)	6.2 (5.3–7.3)	5.8 (4.9–6.9)	
Other races	4.8 (4.0–5.8)	6.0 (4.3–8.2)	5.7 (3.9–8.4)	5.3 (3.6–7.6)	2.4 (1.5–3.8)	
Education						<.001
Less than high school	11.5 (10.6–12.6)	14.5 (12.4–17.0)	13.4 (11.4–15.7)	9.5 (8.0–11.3)	9.0 (7.5–10.8)	
High school or equivalent	28.0 (26.1–29.9)	35.7 (31.7–39.8)	27.5 (23.8–31.5)	26.0 (22.4–29.9)	22.9 (19.7–26.5)	
More than high school	60.4 (58.4–62.4)	49.8 (45.7–54.0)	58.9 (54.8–62.9)	64.5(60.4–68.3)	68.1 (64.3–71.6)	
Not recorded	0.1 (0.0–0.2)	–	0.2 (0.1–0.6)	0.1 (0.0–0.2)	0.0(0.0–0.2)	
Poverty-income ratio						<.001
<1.3	18.4 (17.2–19.8)	22.9 (20.2–25.7)	20.0 (17.2–23.1)	16.3 (14.0–19.0)	14.9 (12.8–17.4)	
1.3–1.8	8.4 (7.6–9.3)	10.0 (8.3–12.1)	8.3 (6.6–10.3)	8.2 (6.7–10.1)	7.0 (5.7–8.6)	
≥1.8	62.8 (60.9–64.6)	57.0 (53.1–60.8)	59.5 (55.5–63.4)	66.6 (62.9–70.1)	67.3 (63.8–70.7)	
Not recorded	10.4 (9.3–11.7)	10.1 (8.1–12.6)	12.2 (9.7–15.2)	8.8 (6.9–11.2)	10.7 (8.6–13.3)	
Alcohol use						<.001
None	7.2 (6.2–8.3)	7.1 (5.2–9.5)	6.6 (5.2–8.5)	6.8 (5.0–9.3)	8.2 (6.3–10.5)	
Moderate	33.3 (31.2–35.4)	28.1 (24.3–32.3)	32.1 (28.1–36.4)	36.3 (32.2–40.8)	36.3 (32.3–40.5)	
Heavy	42.9 (40.8–45.0)	47.6 (43.5–51.8)	46.5 (42.3–50.7)	40.9 (36.7–45.2)	37.2 (33.3–41.3)	
Not recorded	16.6 (15.3–18.1)	17.2 (14.5–20.3)	14.8 (12.5–17.4)	15.9 (13.3–19.0)	18.4 (15.5–21.7)	
BMI						<.001
<25	26.5 (24.6–28.4)	23.1 (19.8–26.8)	21.9 (18.6–25.7)	24.6 (21.1–28.6)	35.5 (31.6–39.6)	
25–30	30.9 (29.0–32.9)	20.3 (17.2–23.7)	30.3 (26.6–34.2)	39.0 (34.8–43.4)	33.8 (29.9–37.8)	
≥30	41.8 (39.7–43.9)	55.5 (51.3–59.5)	47.1 (43.0–51.4)	35.8 (31.8–39.9)	29.9 (26.3–33.8)	
Not recorded	0.8 (0.6–1.1)	1.2 (0.7–2.0)	0.7 (0.4–1.3)	0.6 (0.3–1.2)	0.9 (0.4–1.6)	
Physical activity						<.001
Inactive	46.9 (44.8–49.0)	45.5 (41.4–49.6)	44.4 (40.3–48.6)	43.7 (39.5–48.0)	53.5 (49.3–57.6)	
Less active	8.6 (7.4–9.9)	6.2 (4.6–8.3)	9.4 (7.1–12.5)	9.5 (7.2–12.5)	9.3 (7.1–12.0)	
Active	44.1 (42.0–46.3)	48.1 (43.9–52.2)	45.8 (41.6–50.0)	46.4 (42.1–50.7)	36.7 (32.8–40.8)	
Not recorded	0.4 (0.2–0.8)	0.3 (0.2–0.6)	0.4 (0.2–0.7)	0.4 (0.2–0.9)	0.5 (0.1–3.0)	
Aspirin Use						<.001
Yes	19.0 (17.4–20.7)	17.8 (14.8–21.2)	13.9 (11.3–17.0)	18.8 (15.7–22.4)	24.9 (21.4–28.7)	
Smoking status						<.001
Nonsmoker	58.5 (56.4–60.6)	46.5 (42.3–50.7)	59.0 (54.7–63.2)	61.7 (57.4–65.8)	66.7 (62.6–70.5)	
Former smoker	24.6 (22.8–26.6)	25.9 (22.3–29.9)	24.6 (20.8–28.8)	24.2 (20.6–28.1)	23.8 (20.4–27.6)	
Current smoker	16.8 (15.4–18.4)	27.7 (24.4–31.2)	16.4 (13.7–19.5)	14.1 (11.4–17.3)	9.5 (7.3–12.4)	
CKD						<.001
Yes	15.0 (13.7–16.4)	17.1 (14.5–20.0)	16.5 (13.9–19.5)	10.2 (8.3–12.5)	16.6 (13.8–19.8)	
Albuminuria						.002
Yes	10.0 (9.0–11.2)	11.4 (9.3–13.9)	11.1 (9.2–13.4)	7.4 (5.8–9.3)	10.3 (8.0–13.0)	
Low eGFR						.003
Yes	7.1 (6.2–8.1)	8.2 (6.4–10.3)	7.9 (6.0–10.3)	4.8 (3.5–6.6)	7.5 (5.8–9.6)	

Values are weighted mean ± standard deviation or weighted % (95% confidence interval). P-values are weighted.

UACR: urinary albumin to creatinine ratio; SCr: serum creatinine; eGFR: estimated glomerular filtration rate; hs-CRP: high-sensitivity C-reactive protein; BUN: blood urea nitrogen; SUA: serum uric acid; Hb: hemoglobin; TC: total cholesterol; HDL: high-density lipoprotein; LDL-C: low-density lipoprotein cholesterolemia; VC: vitamin C; BMI: body mass index; CKD: chronic kidney disease.

### Correlation between CKD and vitamin C

As serum levels of vitamin C increased, a discernible reduction in the overall risk of albuminuria, low eGFR, and CKD was observed. Noteworthy was the finding that participants in the third quartile (Q3) group exhibited the lowest prevalence of CKD. To investigate the correlation between serum vitamin C levels and CKD, we employed a weighted multivariable logistic regression analysis, as detailed in Table 2.

**Table 2.** Associations between serum vitamin C level and CKD (*n* = 4969).

	CKD (Yes, *n* = 958)	Model 1 OR (95%CI) *p*	Model 2 OR (95%CI) *p*	Model 3 OR (95%CI) *p*
Quartiles of vitamin C, umol/ L				
Q1(< 30.4)	274	Reference	Reference	Reference
Q2(30.4–50.3)	240	0.96 (0.72, 1.27) .777	0.97 (0.71, 1.32) .843	0.99 (0.71, 1.40) .983
Q3(50.3–67.6)	168	0.55 (0.41, 0.74) <.001	0.51 (0.37, 0.71) <.001	0.53 (0.37, 0.77) .001
Q4(≥ 67.6)	276	0.96 (0.72, 1.29) .806	0.72 (0.52, 0.99) .048	0.75 (0.51, 1.09) .128
P trend	–	<.001	<.001	<.001
Sensitivity analysis after exclusion of participants with dietary vitamin C supplement (None, *n* = 3301)				
Quartiles of vitamin C, umol/ L *N* = 595				
Q1(< 30.4)	247	Reference	Reference	Reference
Q2(30.4–50.3)	174	0.97 (0.71, 1.35) .886	1.05 (0.74, 1.50) .776	1.01 (0.69, 1.48) .971
Q3(50.3–67.6)	90	0.47 (0.33, 0.68) <.001	0.48 (0.33, 0.71) <.001	0.45 (0.29, 0.71) .001
Q4(≥ 67.6)	84	0.67 (0.44, 1.06) .086	0.65 (0.40, 1.04) .073	0.59 (0.37, 0.96) .032
P trend	–	<.001	<.001	<.001

Model 1: unadjusted model; Model 2: minimally adjusted model, adjusted for: gender, age, race; Model 3: fully adjusted model, adjusted for: gender, age, race, hs-CRP, diabetes, hypertension, education, alcohol use, physical activity, aspirin use, smoking, dietary vitamin C intake by food, vitamin C supplement, poverty income ratio, BMI.

CKD: chronic kidney disease; OR: odds ratio; 95% CI: 95% confidence interval.

The association of serum vitamin C levels with a diminished risk of CKD was statistically significant in the Q3 group, evident in both the unadjusted model (OR = 0.55; 95% CI, 0.41–0.74) and the minimally adjusted model (OR = 0.51; 95% CI, 0.37–0.71). In the fully adjusted model, the inverse relationship between serum vitamin C levels and CKD remained stable (OR = 0.53; 95% CI, 0.37–0.77), signifying that participants in the Q3 group had a 46.6% reduced risk of CKD compared to those in the Q1 group. The data in Table 3 elucidate that a significant correlation between serum vitamin C levels and albuminuria was consistently observed in the Q3 group across all models. In the Q3 group, participants exhibited a 37.0% reduced risk of albuminuria compared to those in the Q1 group, with statistical significance (OR = 0.63; 95% CI, 0.43–0.93). Regarding the correlation of serum vitamin C levels with low eGFR, statistically significant associations were noted in the Q3 and Q4 groups in the fully adjusted model (Q3: OR = 0.53; 95% CI, 0.32–0.88; Q4: OR = 0.56; 95% CI, 0.33–0.94), as presented in Table 4.

**Table 3.** Associations between serum vitamin C level and albuminuria (*n* = 4969).

	Albuminuria (Yes, *n* = 681)	Model 1 OR (95%CI) *p*	Model 2 OR (95%CI) *p*	Model 3 OR (95%CI) *p*
Quartiles of vitamin C, umol/ L				
Q1(< 30.4)	196	Reference	Reference	Reference
Q2(30.4–50.3)	173	0.97 (0.71, 1.33) .847	0.92 (0.67, 1.27) .626	0.97 (0.68, 1.37) .858
Q3(50.3–67.6)	129	0.62 (0.44, 0.87) .005	0.59 (0.42, 0.83) .003	0.63 (0.43, 0.93) .021
Q4(≥ 67.6)	183	0.89 (0.62, 1.26) .505	0.76 (0.52, 1.12) .161	0.78 (0.51, 1.20) .252
*p* trend	–	<.001	<.001	<.001
Sensitivity analysis after exclusion of participants with dietary vitamin C supplement (None, *n* = 3301)				
Quartiles of vitamin C, umol/ L *N* = 436				
Q1(< 30.4)	177	Reference	Reference	Reference
Q2(30.4–50.3)	118	0.92 (0.65, 1.30) 0.630	0.87 (0.62, 1.24) .439	0.85 (0.57, 1.27) .432
Q3(50.3–67.6)	71	0.55 (0.36, 0.83) .005	0.53 (0.35, 0.82) .004	0.54 (0.33, 0.88) .014
Q4(≥ 67.6)	70	0.81 (0.49, 1.36) .432	0.77 (0.46, 1.30) .327	0.76 (0.45, 1.27) .289
*p* trend	–	<.001	<.001	<.001

Model 1: unadjusted model; Model 2: minimally adjusted model, adjusted for: gender, age, race; Model 3: fully adjusted model, adjusted for: gender, age, race, hs-CRP, diabetes, hypertension, education, alcohol use, physical activity, aspirin use, smoking, dietary vitamin C intake by food, vitamin C supplement, poverty income ratio, BMI.

OR: odds ratio; 95% CI: 95% confidence interval.

**Table 4.** Associations between serum vitamin C level and low eGFR (*n* = 4969).

	Low eGFR (Yes, *n* = 446)	Model 1 OR (95%CI) *p*	Model 2 OR (95%CI) *p*	Model 3 OR (95%CI) *p*
Quartiles of vitamin C, umol/ L				
Q1(< 30.4)	130	Reference	Reference	Reference
Q2(30.4–50.3)	114	0.96 (0.65, 1.43) .853	1.06 (0.67, 1.69) .792	1.03 (0.61, 1.73) .911
Q3(50.3–67.6)	78	0.57 (0.37, 0.87) .009	0.52 (0.32, 0.82) .005	0.53 (0.32, 0.88) .015
Q4(≥ 67.6)	124	0.91 (0.62, 1.33) .616	0.56 (0.37, 0.84) .006	0.56 (0.33, 0.94) .029
*p* trend	–	<.001	<.001	<.001
Sensitivity analysis after exclusion of participants with dietary vitamin C supplement (None, *n* = 3301)				
Quartiles of vitamin C, umol/ L *N* = 263				
Q1(<30.4)	119	Reference	Reference	Reference
Q2(30.4–50.3)	85	0.99 (0.62,1.58) .979	1.31 (0.75,2.28) .342	1.39 (0.79,2.44) .250
Q3(50.3–67.6)	34	0.39 (0.23,0.64) <.001	0.41 (0.24,0.72) .002	0.43 (0.23,0.81) .010
Q4(≥67.6)	25	0.42 (0.22,0.78) .006	0.40 (0.20,0.78) .007	0.39 (0.19,0.84) .015
*p* trend	–	<.001	<.001	<.001

Model 1: unadjusted model; Model 2: minimally adjusted model, adjusted for: gender, age, race; Model 3: fully adjusted model, adjusted for: gender, age, race, hs-CRP, diabetes, hypertension, education, alcohol use, physical activity, aspirin use, smoking, dietary vitamin C intake by food, vitamin C supplement, poverty income ratio, BMI.

eGFR: estimated glomerular filtration rate; OR: odds ratio; 95% CI: 95% confidence interval.

It is imperative to acknowledge that 33.6% of participants consumed vitamin C supplements, potentially influencing the correlation between serum vitamin C concentrations and these health outcomes. Furthermore, a positive relationship between serum vitamin C concentrations and dietary vitamin C supplements was observed. Therefore, we conducted a sensitivity analysis to assess the robustness of the results. The findings remained consistent with the previously mentioned conclusions, except for the emergence of statistically significant associations between serum vitamin C concentrations and CKD in the Q4 group in the fully adjusted model (Q4: OR = 0.59; 95% CI, 0.37–0.96).

To explore the correlation between CKD and serum vitamin C levels across distinct demographic categories, a subgroup analysis stratified by race, age, and gender was undertaken, as delineated in Supplementary Tables S2–S4. Upon gender stratification, it was observed that both men and women in the Q3 group exhibited a decreased risk of CKD, with a 48.8% reduction in risk for men and a 40.1% reduction for women, respectively. Notably, serum vitamin C concentrations were relatively lower in men (fully adjusted model: Q3, OR = 0.47; 95% CI, 0.24–0.91) compared to women (fully adjusted model: Q4, OR = 0.40; 95% CI, 0.19–0.87), and this difference was inversely associated with low eGFR. When data were stratified by age, results indicated that individuals in the age group of 65-80 exhibited significantly reduced risks of both CKD (fully adjusted model: Q3, OR = 0.41, 95% CI, 0.25–0.69; Q4, OR = 0.53, 95% CI, 0.31–0.91) and low eGFR (fully adjusted model: Q3, OR = 0.44, 95% CI, 0.24-0.79; Q4, OR = 0.52, 95% CI, 0.27–0.98) in the Q3–Q4 group. Furthermore, the subgroup analysis unveiled that Non-Hispanic White participants in the Q3 group were associated with a significantly reduced risk of CKD (fully adjusted model: OR = 0.54, 95% CI, 0.31–0.94), while Hispanic participants in the Q3 group were correlated with a remarkably reduced risk of low eGFR (fully adjusted model: OR = 0.28; 95% CI, 0.09–0.86). These findings illuminate the varying degrees of association within these distinct subgroups, underscoring the multifaceted nature of the correlation between CKD and serum vitamin C concentrations.

## Discussion

In this study encompassing 4,969 participants, a statistically significant correlation between serum vitamin C concentrations and the risk of CKD was observed. Higher levels of serum vitamin C were associated with a reduced likelihood of CKD. Specifically, individuals in the Q3 group of serum vitamin C concentration demonstrated a significantly diminished risk of albuminuria, low eGFR, and CKD. Furthermore, our analysis indicated that elevated serum vitamin C levels in the elderly were linked to a substantial reduction in the risk of developing CKD and a decline in eGFR.

It is crucial to recognize that research on the correlation between serum vitamin C concentrations and CKD has been limited. A cohort study conducted as part of the Tehran Lipid and Glucose Study demonstrated that an increased intake of several micronutrients, including vitamin C, was correlated with a reduced CKD risk [27]. However, the utilization of information about dietary vitamin C data from a food-frequency questionnaire in that study may introduce significant retrospective bias, potentially compromising the reliability of the results. In contrast, serum vitamin C levels offer a more robust assessment of tissue vitamin C, providing valuable insights into its functions in disease prevention [28]. Numerous studies have underscored the close relationship between serum vitamin C concentration and the risk of various chronic diseases, including heart failure [29], cancer [30,31], cognitive dysfunction [32], and Alzheimer’s disease [33]. A prior study in Japan observed a positive linear correlation between serum vitamin C concentration and eGFR [34]. Although this study is prospective, it featured a relatively small sample size, comprising only 58 outpatients and focusing on CKD stages 3-5. Our population-based findings, indicating a CKD prevalence of 15.0%, align fundamentally with the estimated 14.5% for the American population in 2017, as reported by the American Kidney Data System in 2019 [35]. Our study revealed that individuals in the Q3 group, characterized by higher serum vitamin C levels, experienced a significant decrease in the risk of CKD, including the risk of albuminuria and reduced eGFR. The serum vitamin C levels in the Q3 group ranged from 50.3 to 67.6 mmol/L, a range consistent with the optimal serum level for health, estimated to be approximately 70 mmol/L in prior research [36]. An interesting observation was that, while the highest quartile of vitamin C was correlated with a risk of reduced eGFR, it did not correspond to a risk of albuminuria. This complexity can be attributed to the dual nature of vitamin C. On one hand, high-dose vitamin C administration may act as a pro-oxidant rather than an antioxidant [37]. On the other hand, high doses of vitamin C could lead to the accumulation of its metabolites and oxalates, potentially resulting in kidney stones and tissue damage [38]. Findings from a randomized clinical trial supported the idea that vitamin C and E supplementation can protect renal function by reducing urinary albumin and improving glomerular function in individuals with type 2 diabetes [39]. Moreover, evidence has indicated that antioxidant therapy with bardoxolone methyl significantly lowers serum creatinine levels and elevates eGFR levels in CKD patients, compared to a placebo group [40]. Animal studies consistently demonstrated that vitamin C can ameliorate acute kidney injury (AKI) by reducing lipid oxidation and inflammation, as well as partially enhancing oxygen supply and consumption in the kidney [41–43]. In a rat model, only rats supplemented with vitamin C were protected from the effects of kidney failure. Vitamin C inhibited oxidative stress and protected renal function by reducing urinary protein, plasma urate levels, and renal casts in the renal tubules [44]. This aligns with our study’s results, indicating that higher serum vitamin C concentration correlates with lower levels of urine protein and plasma uric acid. Furthermore, our stratified analysis revealed that elderly individuals with higher serum vitamin C levels exhibited a reduced risk of CKD and low eGFR, a finding of substantial clinical significance. Oxidative stress is increasingly recognized as the main epigenetic factor of aging. As the aging process progresses, the endogenous antioxidant defense system may significantly decrease, while vitamin C acts as a potent antioxidant [45]. Given that the prevalence of CKD significantly increases with age [46], our findings hold promise for guiding the administration of appropriate vitamin C supplementation in the elderly to protect their renal function.

Aligned with prior research, our study emphasized that individuals with reduced serum vitamin C levels were predominantly male, possessed lower educational attainment, exhibited a diminished PIR, and partook in current smoking [47]. Prior studies have consistently demonstrated an independent association between smoking and a decrease in circulating vitamin C concentrations. Specifically, circulating levels of vitamin C, α-carotenoids, β-carotene, and cryptoxanthin are, on average, more than 25% lower in active smokers compared to nonsmokers [48]. Additionally, our study identified a decline in serum vitamin C concentration among heavy drinkers, notwithstanding the absence of a clearly defined link between excessive alcohol consumption and vitamin C deficiency in existing research [47]. This observation merits further scrutiny. Furthermore, our study observed that individuals with diabetes and obesity were inclined to exhibit lower serum vitamin C concentrations. This correlation is understandable, given that obesity is a recognized risk factor prevalent among CKD patients [49]. Additionally, diabetes often leads to microvascular complications, including diabetic nephropathy, a key etiology of CKD [50]. Multiple studies have established markedly lower serum vitamin C concen­trations in individuals with diabetes compared to their non-diabetic counterparts. Contributing factors may include restricted dietary choices and/or intake, along with increased oxidative stress associated with the development of diabetes [51].

A notable strength of our study lies in its nationwide, population-based sampling survey, ensuring a substantial and appropriately weighted sample size for enhanced representativeness. Moreover, our study rigorously adjusted for confounding factors and conducted sensitivity analyses, augmenting the reliability of our findings. Nevertheless, our study does bear certain limitations. Firstly, due to its cross-sectional nature, it cannot definitively establish the causal correlation between serum vitamin C levels and the onset of CKD. Secondly, our reliance on a single measurement of serum vitamin C renders our study susceptible to random errors. Additionally, the assessment of renal function was confined to a specific point in time, neglecting anomalies under physiological conditions or those arising from AKI. Moreover, certain potential confounders, such as dialysis treatment and the use of medications like diuretics or multi-supplements, which may influence serum vitamin C concentrations, were not accounted for in our analysis.

## Conclusion

In conclusion, the findings from our study strongly indicate a correlation between elevated serum vitamin C concentrations and a reduced risk of developing albuminuria, low eGFR, and CKD. This suggests that judiciously augmenting serum vitamin C levels could potentially mitigate the risk of CKD, especially in the elderly. Furthermore, the scientific community would derive substantial value from comprehensive prospective cohort studies to further investigate the potential therapeutic implications of vitamin C in the context of CKD.

## Supplementary Material

Supplemental Material

## Data Availability

Publicly available datasets were analyzed in this study. These data can be found here: https://www.cdc.gov/nchs/nhanes/.
