# Supplementary material for: Serum vitamin C levels and their correlation with chronic kidney disease in adults: a nationwide study
Source: Ren Fail. 2024 Jan 8;46(1):2298079. doi: 10.1080/0886022X.2023.2298079 (PMC10776057; doi:10.1080/0886022X.2023.2298079)
Supplement: Supplemental Material [file IRNF_A_2298079_SM4623.pdf]

Supplementary Table 1: Associations of Covariates with Kidney Outcomes (N = 4,969)

	CKD	Albuminuria	Low eGFR
Gender (%)			
Male	reference	reference	reference
Female	-0.02 (0.85, 1.14)	-0.05 (0.81, 1.12)	-0.07 (0.76, 1.14)
Age (years)			
18-44	reference	reference	reference
45-64	0.80 (1.76, 2.82)*	0.76 (1.67, 2.72)*	1.44 (2.22, 8.11)*
65-80	1.92 (5.62, 8.34)*	1.35 (3.14, 4.77)*	3.64 (21.75, 66.29)*
Race (%)			
Hispanic	reference	reference	reference
Non-Hispanic White	0.37 (1.18, 1.75)*	0.02 (0.82, 1.27)	1.05 (2.07, 3.99)*
Non-Hispanic Black	0.47 (1.30, 1.99)*	0.09 (0.86, 1.39)	1.31 (2.65, 5.23)*
Non-Hispanic Asian	-0.11 (0.69, 1.17)	-0.01 (0.76, 1.31)	-0.38 (0.40, 1.17)
Other races	0.19 (0.85, 1.72)	0.13 (0.78, 1.66)	0.15 (0.60, 2.21)
Education			
Less than high school	reference	reference	reference
High school or equivalent	-0.20（0.67, 1.01)	-0.30（0.59, 0.93)*	-0.04（0.72, 1.28)
More than high school	-0.35（0.59, 0.84)*	-0.42（0.54, 0.80)*	-0.22（0.62,1.04)
Smoking status			
Nonsmoker	reference	reference	reference
Former smoker	0.57 (1.51, 2.09)*	0.31 (1.13, 1.65)*	0.95 (2.08, 3.23)*
Current smoker	0.01 (0.82, 1.23)	0.03 (0.83, 1.30)	-0.12 (0.64, 1.23)
Alcohol use			
None	reference	reference	reference
Moderate	-0.23 (0.62, 1.03)	-0.50 (0.46, 0.80)*	0.15 (0.78, 1.71)
Heavy	-0.46 (0.49, 0.81)*	-0.54 (0.45, 0.76)*	-0.39 (0.45, 1.01)
Diabetes (%)			
no	reference	reference	reference
yes	1.40 (3.48, 4.75)*	1.52 (3.84, 5.41)*	1.08 (2.39, 3.64)*
Hypertension (%)			
no	reference	reference	reference

Information Classification: General

yes	1.37 (3.31, 4.70)*	1.10 (2.47, 3.62)*	2.20 (6.32, 12.76)*
Aspirin Use			
no	reference	reference	reference
yes	1.14 (2.69, 3.66)*	0.92 (2.11, 2.99)*	1.34 (3.10, 4.69)*
Poverty-income ratio			
＜1.3	reference	reference	reference
1.3-1.8	0.15 (0.92, 1.48)	0.04 (0.79, 1.36)	0.26 (0.93, 1.81)
≥1.8	-0.10 (0.76, 1.08)	-0.23 (0.65, 0.96)*	0.01 (0.78, 1.29)
BMI			
＜25	reference	reference	reference
25-30	0.15 (0.95, 1.42)	-0.04 (0.77, 1.20)	0.45 (1.17, 2.12)*
≥30	0.44 (1.29, 1.87)*	0.30 (1.10, 1.65)*	0.63 (1.41, 2.48)*
Dietary VC intake by food, mg/d			
＜25.6	reference	reference	reference
25.6-56.7	0.02 (0.83, 1.26)	0.13 (0.89, 1.45)	-0.08 (0.69, 1.24)
56.7-106.7	0.02 (0.83, 1.27)	0.12 (0.89, 1.44)	-0.30 (0.55, 1.01)
≥106.7	0.02 (0.83, 1.26)	0.11 (0.88, 1.43)	-0.22 (0.59, 1.08)
Daily dose of VC supplements, mg			
None	reference	reference	reference
1-60	0.85 (1.70, 3.22)*	0.65 (1.32, 2.76)*	1.10 (1.89, 4.75)*
61-120	0.73 (1.45, 2.95)*	0.63 (1.26, 2.82)*	0.83 (1.36, 3.84)*
121-500	0.89 (1.68, 3.51)*	0.79 (1.44, 3.34)*	0.89 (1.42, 4.19)*
≥500	0.37 (1.11, 1.89)*	0.37 (1.07, 1.95)*	0.43 (1.03, 2.33)*
Physical activity			
Inactive	reference	reference	reference
Less active	-0.01 (0.77, 1.29)	-0.13 (0.65, 1.19)	0.16 (0.84, 1.65)
Active	-0.44 (0.55, 0.75)*	-0.42 (0.55, 0.79)*	-0.57 (0.45, 0.71)*
hs-CRP, mg/L	0.02 (1.01, 1.03)*	0.02 (1.02, 1.03)*	0.01 (1.00, 1.02)*

Values are regression coefficients (95% Confidence Interval) from univariate regression models and reflect differences in kidney outcomes per unit change of each covariate and for different categories of each covariate as compared to the reference group. *P-value <0.05.

CKD, chronic kidney disease; eGFR, estimated glomerular filtration rate; hs-CRP, high-sensitivity C-reactive protein; VC, vitamin C; BMI, body mass index.

Information Classification: General

Supplementary Table 2: Subgroup analysis of associations between serum vitamin C level and CKD (n=4969)

	Fully adjusted model OR (95%CI), *P*			
	Q1(< 30.4)	Q2(30.4-50.3)	Q3(50.3-67.6)	Q4(≥ 67.6)
Stratified by gender				
Male	Reference	1.23（0.77, 1.97) 0.387	0.51（0.31, 0.85) 0.009	0.86（0.51, 1.46) 0.571
Female	Reference	0.91（0.58, 1.44) 0.689	0.60（0.36, 0.99) 0.044	0.68（0.40, 1.16) 0.155
Stratified by age (years)				
18-45	Reference	0.95（0.50, 1.80) 0.874	0.79（0.37, 1.66) 0.532	1.06（0.53, 2.11) 0.866
45-65	Reference	1.14（0.57, 2.29) 0.709	0.51（0.25, 1.03) 0.059	0.71（0.31, 1.58) 0.395
65-80	Reference	0.87（0.52, 1.44) 0.579	0.41（0.25, 0.69) 0.001	0.53（0.31, 0.91) 0.020
Stratified by race				
Hispanic	Reference	0.98（0.56, 1.72) 0.939	0.51（0.26, 1.02) 0.058	0.52（0.25, 1.09) 0.085
Non-Hispanic White	Reference	1.09（0.64, 1.87) 0.748	0.54（0.31, 0.94) 0.029	0.78（0.44, 1.37) 0.387
Non-Hispanic Black	Reference	1.02（0.62, 1.69) 0.929	0.59（0.34, 1.05) 0.073	0.61（0.34, 1.10) 0.101
Non-Hispanic Asian	Reference	1.64（0.68, 3.94) 0.270	0.99（0.40, 2.51) 0.998	0.91（0.36, 2.30) 0.838
Other races	Reference	0.04（0.01, 0.25) 0.001	0.15（0.04, 0.58) 0.006	0.92（0.24, 3.53) 0.907

Note: fully adjusted model, adjusted for: gender, age, race, hs-CRP, diabetes, hypertension, education, alcohol use, physical activity, aspirin use, smoking, dietary vitamin C intake by food, vitamin C supplement, poverty income ratio, BMI.

Abbreviations: CKD, chronic kidney disease; OR, odds ratio; 95% CI, 95% confidence interval

Information Classification: General

Supplementary Table 3: Subgroup analysis of associations between serum vitamin C level and albuminuria (n=4969)

	Fully adjusted model OR (95%CI), *P*			
	Q1(< 30.4)	Q2(30.4-50.3)	Q3(50.3-67.6)	Q4(≥ 67.6)
Stratified by gender				
Male	Reference	1.15（0.70, 1.90) 0.573	0.72（0.42, 1.24) 0.238	0.68（0.38, 1.20) 0.180
Female	Reference	0.87（0.54, 1.41) 0.574	0.59（0.34, 1.03) 0.061	0.80（0.44, 1.46) 0.471
Stratified by age (years)				
18-45	Reference	0.99（0.50, 1.93) 0.965	0.80（0.37, 1.73) 0.577	1.09（0.54, 2.22) 0.808
45-65	Reference	0.79（0.40, 1.57) 0.505	0.36（0.17, 0.74) 0.006	0.75（0.32, 1.74) 0.500
65-80	Reference	1.09（0.64, 1.85) 0.756	0.71（0.39, 1.28) 0.255	0.55（0.29, 1.04) 0.068

Information Classification: General

Stratified by race				
Hispanic	Reference	1.14（0.63, 2.09) 0.662	0.59（0.28, 1.22) 0.151	0.52（0.23, 1.18) 0.116
Non-Hispanic White	Reference	0.94（0.51, 1.74) 0.836	0.70（0.36, 1.35) 0.285	0.84（0.43, 1.66) 0.621
Non-Hispanic Black	Reference	1.14（0.66, 1.98) 0.638	0.58（0.30, 1.11) 0.098	0.61（0.32, 1.15) 0.125
Non-Hispanic Asian	Reference	1.45（0.59, 3.56) 0.417	1.03（0.41, 2.58) 0.955	0.81（0.31, 2.09) 0.662
Other races	Reference	0.06（0.01, 0.37) 0.003	0.18（0.05, 0.65) 0.009	1.26（0.32, 4.95) 0.742

Note: fully adjusted model, adjusted for: gender, age, race, hs-CRP, diabetes, hypertension, education, alcohol use, physical activity, aspirin use, smoking, dietary vitamin C intake by food, vitamin C supplement, poverty income ratio, BMI.

Abbreviations: OR, odds ratio; 95% CI, 95% confidence interval.

Supplementary Table 4: Subgroup analysis of associations between serum vitamin C level and low eGFR (n=4969)

Fully adjusted model OR (95%CI), *P*

	Q1 (＜30.4)	Q2 (30.4-50.3)	Q3 (50.3-67.6)	Q4 (≥67.6)
Stratified by gender				
Male	Reference	1.20（0.60, 2.38) 0.604	0.47（0.24, 0.91) 0.026	0.70（0.33, 1.47) 0.346
Female	Reference	0.93（0.44, 1.95) 0.849	0.57（0.27, 1.20) 0.138	0.40（0.19, 0.87) 0.020
Stratified by age (years)				
18-45	Reference	0.25（0.04, 1.44) 0.120	0.23（0.01, 3.79) 0.302	-
45-65	Reference	2.16（0.69, 6.79) 0.187	1.41（0.43, 4.58) 0.569	0.19（0.03, 1.14) 0.070
65-80	Reference	0.82（0.46, 1.45) 0.490	0.44（0.24, 0.79) 0.006	0.52（0.27, 0.98) 0.044
Stratified by race				
Hispanic	Reference	0.66（0.27, 1.64) 0.374	0.28（0.09, 0.86) 0.027	0.39（0.11, 1.33) 0.132
Non-Hispanic White	Reference	1.17（0.57, 2.39) 0.665	0.57（0.29, 1.12) 0.104	0.52（0.26, 1.05) 0.067
Non-Hispanic Black	Reference	0.76（0.39, 1.46) 0.410	0.45（0.19, 1.05) 0.065	0.84（0.36, 1.98) 0.693
Non-Hispanic Asian	Reference	6.04（0.78, 46.52) 0.085	0.80（0.10, 6.65) 0.836	1.33（0.18, 9.75) 0.778
Other races	Reference	0.02（0.00, 2.50) 0.108	0.30（0.01, 9.54) 0.492	0.23（0.00, 109.73) 0.641

Note: fully adjusted model, adjusted for: gender, age, race, hs-CRP, diabetes, hypertension, education, alcohol use, physical activity, aspirin use, smoking, dietary vitamin C intake by food, vitamin C supplement, poverty income ratio, BMI.

Abbreviations: OR, odds ratio; 95% CI, 95% confidence interval; eGFR, estimated glomerular filtration rate.

Information Classification: General
